# The prognostic correlation of AFP level at diagnosis with pathological grade, progression, and survival of patients with hepatocellular carcinoma

**DOI:** 10.1038/s41598-017-12834-1

**Published:** 2017-10-09

**Authors:** Dou-Sheng Bai, Chi Zhang, Ping Chen, Sheng-Jie Jin, Guo-Qing Jiang

**Affiliations:** Department of Hepatobiliary Surgery, Clinical Medical College of Yangzhou University, Yangzhou, 225001 China

## Abstract

The purpose of this study was to conduct a comprehensive study of the clinical correlation between the alpha-fetoprotein (AFP) level at diagnosis and pathological grades, progression, and survival of patients with hepatocellular carcinoma (HCC). A total of 78,743 patients in Surveillance, Epidemiology, and End Results Program (SEER)-registered HCC was analyzed. The AFP test results for patients with HCC were mainly recorded as AFP-negative and AFP-positive. Logistic regression analysis revealed that the AFP level at diagnosis was an independent risk factor of pathological grade (odds ratio [OR], 2.559; 95% confidence interval [CI], 2.075–3.157; *P* < 0.001), TNM-7 stage (OR, 2.794; CI, 2.407–3.242; *P* < 0.001), and tumor size (OR, 1.748; 95% CI, 1.574–1.941; *P* < 0.001). Multivariable Cox regression analyses identified AFP level as an independent predictor of survival risk of patients with HCC who did not undergo surgery (hazard ratio [HR], 1.660; 95% CI, 1.534–1.797; *P* < 0.001), and those who underwent surgery (HR, 1.534; 95% CI, 1.348–1.745; *P* < 0.001). The AFP level at diagnosis was an independent risk predictor associated with pathological grade, progression, and survival. Further, surgery may not significantly reverse the adverse effects of AFP-positive compared with AFP-negative.

## Introduction

Hepatocellular carcinoma (HCC) is the sixth most prevalent tumor worldwide and the third leading cause of cancer-related death^1^. More than 250,000 new cases of HCC occur annually, and approximately 500,000–600,000 patients die each year^2,3^. It is therefore critically important to identify factors that correlate with pathological grade, progression, and prognosis of survival. Serum alpha-fetoprotein (AFP) is the most widely used serological marker to establish a diagnosis of HCC and was included in international guidelines for HCC surveillance^4–6^. Approximately 50% of HCCs secrete AFP^7,8^, and a plasma AFP concentration >400 ng/ml is generally considered a reliable for supporting the diagnosis of HCC. Because the accuracy of the AFP concentration was challenged as well as a growing debate about its ongoing use for HCC surveillance programs, AFP was removed from updated international guidelines for HCC surveillance. However, many reports suggest a rationale for the continued use of AFP^9–11^.

To identify the clinical significance of AFP levels, in the present study we analyzed the comprehensive clinical relationship between the AFP levels and pathological grades, progression, and survival of patients with HCC using the data for patients with HCC that were deposited in the cancer registry of the Surveillance, Epidemiology, and End Results (SEER) Program from 1988 through 2013.

## Results

### Baseline Patient Characteristics

A comparative analysis of baseline demographics and tumor characteristics of the AFP-negative and AFP-positive groups revealed that the former included higher proportions of patients≥60 years of age (62.2%), White (73.6%) and married (56.7%) patients, well/moderately differentiated tumors (85.9%), TNM-7-stage I/II tumors (76.5%), tumors ≤5 cm in diameter (64.4%), and F0 tumors (22.8%) (all *P* < 0.05). The proportions of men and women did not differ significantly in the two groups (Table 1).

**Table 1:** Baseline demographic and tumor characteristics of patients with AFP-negative and AFP-positive.

Characteristic	Total	AFP		*P*
		Negative	Positive	
	*N*	*N* (%)	*N* (%)	
Sex	38820			0.057
Male		7172 (78.1)	22872 (77.2)	3.610
Female		2009 (21.9)	6767 (22.8)	
Age	38820			<0.001
<60		3466 (37.8)	13069 (44.1)	115.306
≥60		5715 (62.2)	16570 (55.9)	
Race	38674			<0.001
White		6731 (73.6)	19273 (65.3)	313.483
Black		802 (8.8)	4604 (15.6)	
Other*		1610 (17.6)	5654 (19.1)	
Pathological grade	14007			<0.001
Well/Moderate		3587 (85.9)	7026 (71.5)	334.795
Poor/Anaplastic		587 (14.1)	2807 (28.5)	
TNM 7 Stage	17102			<0.001
I/II		3403 (76.5)	6892 (54.5)	668.522
III/IV		1044 (23.5)	5763 (45.5)	
Tumor Size	32714			<0.001
≤5 cm		5343 (64.4)	12788 (52.4)	360.653
>5 cm		2957 (35.6)	11626 (47.6)	
Fibrosis	10714			<0.001
F0^†^		629 (22.8)	1367 (17.2)	43.184
F1^††^		2126 (77.2)	6592 (82.8)	
Marital Status	37381			<0.001
Married		5012 (56.7)	14897 (52.2)	53.577
Non-married^#^		3835 (43.3)	13637 (47.8)	

*Other includes American Indian/Alaskan native, and Asian/Pacific Islander.

^†^F0, equivalent to Ishak score 0–4.

^††^F1, equivalent to Ishak score 5–6.

^#^Non-married includes widowed, never married, divorced, separated, unmarried and domestic Partner.

### Association between AFP levels and pathological grades

**Figure 1:**
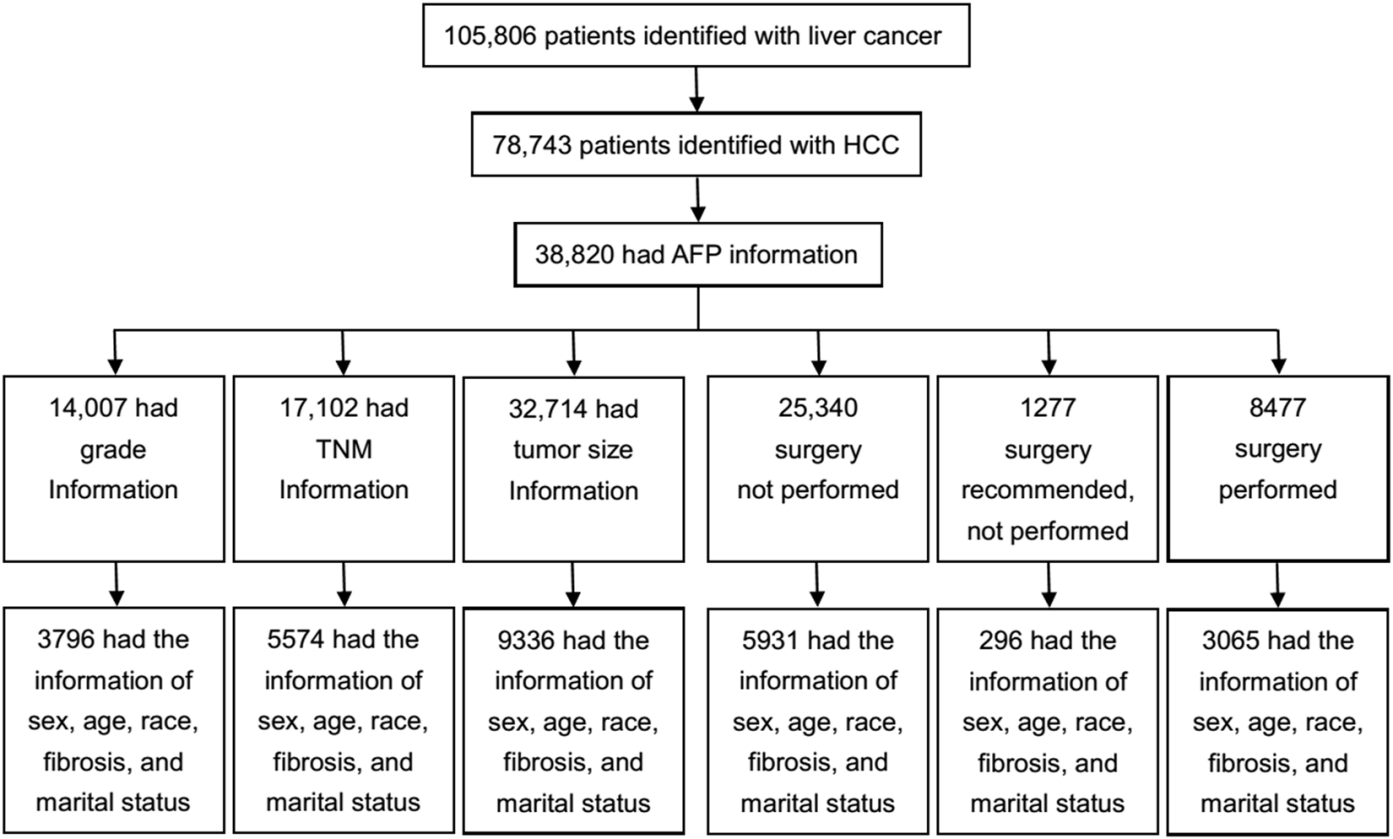
CONSORT diagram. Patients with HCC and the required clinical data between 1988 and 2013 were selected from the cancer registries of the Surveillance, Epidemiology, and End Results Program.

From an initial sample of 105,806 patients identified with liver cancer, 3796 fulfilled our eligibility criteria for evaluating the association of AFP levels with pathological grades (Fig. 1). There were lower proportions of other races (19.6%), “F0” tumors (28.8%), and AFP-positive tumors (66.0%) in patients with well/moderately differentiated tumors compared with those with poorly differentiated/anaplastic tumors (all *P* < 0.05). Logistic regression analysis revealed that the AFP level was an independent predictor of pathological grade (odds ratio [OR], 2.559; 95% confidence interval [CI], 2.075 to 3.157; *P* < 0.001) (Table 2). Further, poorly differentiated/anaplastic tumors were significantly more prevalent in the AFP-positive group compared with those in the AFP-negative group (22.8% vs 10.5%, *P* < 0.001). Moreover, 83.0% of tumors were AFP-positive in the poorly differentiated/anaplastic group, whereas 66.0% were classified as AFP-positive in the well/moderately group (*P* < 0.001) (Table 2).

**Table 2:** Univariate and logistic multivariable regression analysis of the association of AFP levels with tumor cell differentiation.

Variable	pathological grade		χ^2^ test	*P*	logistic regression analysis	*P*
	Well/Moderate	Poor/Anaplastic				
	**(n = 3073)** ***N*** **(%)**	**(n = 723)** ***N*** **(%)**				
Sex			0.151	0.698		NI
Male	2388 (77.7)	557 (77.0)				
Female	685 (22.3)	166 (23.0)				
Age			<0.001	0.992		NI
<60	1365 (44.4)	321 (44.4)				
≥60	1708 (55.6)	402 (55.6)				
Race			31.811	<0.001		<0.001
White	2102 (68.4)	430 (59.5)			Reference	
Black	370 (12.0)	83 (11.5)			0.983 (0.756–1.277)	0.895
Other*	601 (19.6)	210 (29.0)			1.633 (1.346–1.980)	<0.001
Fibrosis			4.767	0.029	0.822 (0.687–0.984)	0.032
F0^†^	885 (28.8)	238 (32.9)				
F1^††^	2188 (71.2)	485 (67.1)				
AFP			79.079	<0.001	2.559 (2.075–3.157)	<0.001
Negative	1044 (34.0)	123 (17.0)				
Positive	2029 (66.0)	600 (83.0)				
Marital Status			0.028	0.867		NI
Married	1847 (60.1)	437 (60.4)				
Non-married^#^	1226 (39.9)	286 (39.6)				

^*^Other includes American Indian/Alaska native, and Asian/Pacific Islander.

^†^F0, equivalent to Ishak score 0–4.

^††^F1, equivalent to Ishak score 5–6.

^#^Non-married includes widowed, never married, divorced, separated, unmarried, and domestic partner. NI: not included in the logistic multivariable regression analysis.

### Association between AFP levels and TNM-7 stage (HCC progression)

The eligibility criteria for evaluating the association of AFP levels on TNM-7 stage were met by 5574 patients (Fig. 1). There were greater proportions of women (*P* < 0.001), Whites (*P* < 0.001), F1 tumors (*P* = 0.003), AFP-negative tumors (*P* < 0.001), and married patients (*P* = 0.002) among patients with TNM-7 stage I/II tumors compared with those with TNM-7-stage III/IV tumors (Table 3). Logistic regression analysis revealed that the AFP level was an independent predictor of TNM-7 stage (OR, 2.794; 95% CI, 2.407–3.242; *P* < 0.001) (Table 3). Further, TNM-7-stage I/II tumors were significantly more prevalent in the AFP-negative group with those in the AFP-positive group (82.8% vs 63.8%, *P* < 0.001). Compared with patients with TNM-stage I/II, patients with TNM-7-stage III/IV had a higher proportion of tumors associated with AFP-positive (*P* < 0.001) (Table 3).

**Table 3:** Univariate and logistic multivariable regression analysis of the association of AFP level with TNM-7 stage.

Variable	TNM-7 Stage		χ^2^ test	*P*	logistic regression analysis	*P*
	I/II	III/IV				
	**(n = 3853)** ***N*** **(%)**	**(n = 1721)** ***N*** **(%)**				
Sex			31.659	<0.001	0.626 (0.540–0.725)	<0.001
Male	2908 (75.5)	1416 (82.3)				
Female	945 (24.5)	305 (17.7)				
Age			2.712	0.100		NI
<60	1575 (40.9)	744 (43.2)				
≥60	2278 (59.1)	977 (56.8)				
Race			14.397	<0.001		0.141
White	2721 (70.6)	1148 (66.7)			Reference	
Black	454 (11.8)	264 (15.3)			1.177 (0.992–1.398)	0.062
Other*	678 (17.6)	309 (18.0)			1.082 (0.925–1.266)	0.326
Fibrosis			8.783	0.003	0.718 (0.615–0.839)	<0.001
F0^†^	598 (15.5)	322 (18.7)				
F1^††^	3255 (84.5)	1399 (81.3)				
AFP			189.206	<0.001	2.794 (2.407–3.242)	<0.001
Negative	1284 (33.3)	266 (15.5)				
Positive	2569 (66.7)	1455 (84.5)				
Marital Status			9.558	0.002	1.193 (1.059–1.344)	0.004
Married	2071 (53.8)	848 (49.3)				
Non-married^#^	1782 (46.2)	873 (50.7)				

*Other includes American Indian/Alaska native, and Asian/Pacific Islander.

^†^F0, equivalent to Ishak score 0–4.

^††^F1, equivalent to Ishak score 5–6.

^#^Non-married includes widowed, never married, divorced, separated, unmarried, and domestic partner. NI: not included in the logistic multivariable regression analysis.

### Association between AFP levels and tumor size (HCC progression)

The eligibility criteria for evaluating the association of AFP levels with tumor size were met by 9336 patients (Fig. 1). The population of patients with tumors ≤5 cm included a greater proportion of women (*P* < 0.001) and patients aged <60 years (*P* = 0.002), more Whites (*P* < 0.001), a greater proportion of tumors associated with F1 fibrosis (*P* < 0.001), and AFP-negative tumors (*P* < 0.001) compared with patients with tumors >5 cm (Table 4). Logistic regression analysis identified the AFP level as an independent predictor of tumor size (OR, 1.748; 95% CI, 1.574–1.941; *P* < 0.001) (Table 4). Further, tumors ≤5 cm were significantly more prevalent in the AFP-negative group compared with those in the AFP-positive group (74.0% % vs 63.7%; *P* < 0.001) (Table 4).

**Table 4:** Univariate and logistic multivariable regression analysis of the association of AFP level with tumor size.

Variable	tumor size		χ^2^ test	*P*	logistic regression analysis	*P*
	≤5 cm	>5 cm				
	**(n = 6207)** ***N*** **(%)**	**(n = 3129)** ***N*** **(%)**				
Sex			13.567	<0.001	0.769 (0.690–0.857)	<0.001
Male	4740 (76.4)	2495 (79.7)				
Female	1467 (23.6)	634 (20.3)				
Age			9.338	0.002	1.159 (1.060–1.267)	0.001
<60	2901 (46.7)	1358 (43.4)				
≥60	3306 (53.3)	1771 (56.6)				
Race			21.081	<0.001		0.050
White	4330 (69.8)	2037 (65.1)			Reference	
Black	703 (11.3)	399 (12.8)			1.101 (0.961–1.263)	0.167
Other*	1174 (18.9)	693 (22.1)			1.136 (1.017–1.270)	0.024
Fibrosis			227.755	<0.001	0.434 (0.390–0.484)	<0.001
F0^†^	890 (14.3)	852 (27.2)				
F1^††^	5317 (85.7)	2277 (72.8)				
AFP			85.791	<0.001	1.748 (1.574–1.941)	<0.001
Negative	1854 (29.9)	653 (20.9)				
Positive	4353 (70.1)	2476 (79.1)				
Marital Status			0.772	0.380		NI
Married	3410 (54.9)	1689 (54.0)				
Non-married^#^	2797 (45.1)	1440 (46.0)				

*Other includes American Indian/Alaska native, and Asian/Pacific Islander.

^†^F0, equivalent to Ishak score 0–4.

^††^F1, equivalent to Ishak score 5–6.

^#^Non-married includes widowed, never married, divorced, separated, unmarried, and domestic partner. NI: not included in the logistic multivariable regression analysis.

### Association between AFP levels and survival

**Figure 2:**
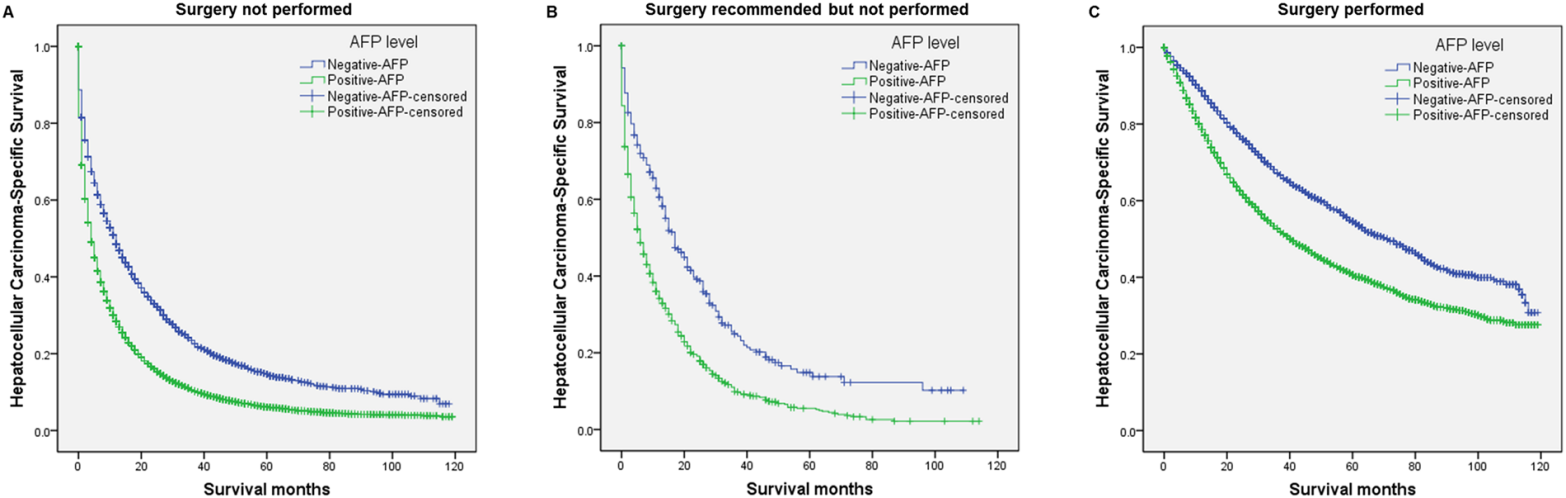
Survival curves based on Kaplan-Meier analysis according to AFP level. Survival curves based on Kaplan–Meier analysis that compare the association of “negative-AFP” and “positive-AFP” with HCC cause-specific survival of (**A**) patients who had not undergone surgery, (**B**) patients for whom surgery was recommended but not performed, and (**C**) patients who had undergone surgery.

Analysis of the association between AFP levels and HCSS of 25,340 patients who did not undergo surgery and met the initial eligibility criteria, revealed that patients with AFP-negative had better 1-year, 3-year, and 5-year HCSS (*P* < 0.001) compared with those with AFP-positive (Fig. 2A and Table 5).

**Table 5:** Kaplan-Meier analysis of the association of AFP-negative and AFP-positive on survival.

Variable	Total	1-year HCCS	3-year HCCS	5-year HCCS	Log rank χ^2^ test	*P*
AFP (surgery not performed)	25340				714.746	<0.001
Negative	4913	48.5%	23.5%	14.7%		
Positive	20427	28.4%	10.6%	6.1%		
AFP (surgery recommended but not performed)	1277				56.788	<0.001
Negative	278	60.6%	24.9%	14.8%		
Positive	999	34.2%	9.9%	5.5%		
AFP (surgery performed)	8477				140.656	<0.001
Negative	2813	88.6%	67.2%	54.6%		
Positive	5664	78.6%	52.7%	40.6%		

Abbreviations: HCSS, hepatocellular carcinoma-specific survival.

**Figure 3:**
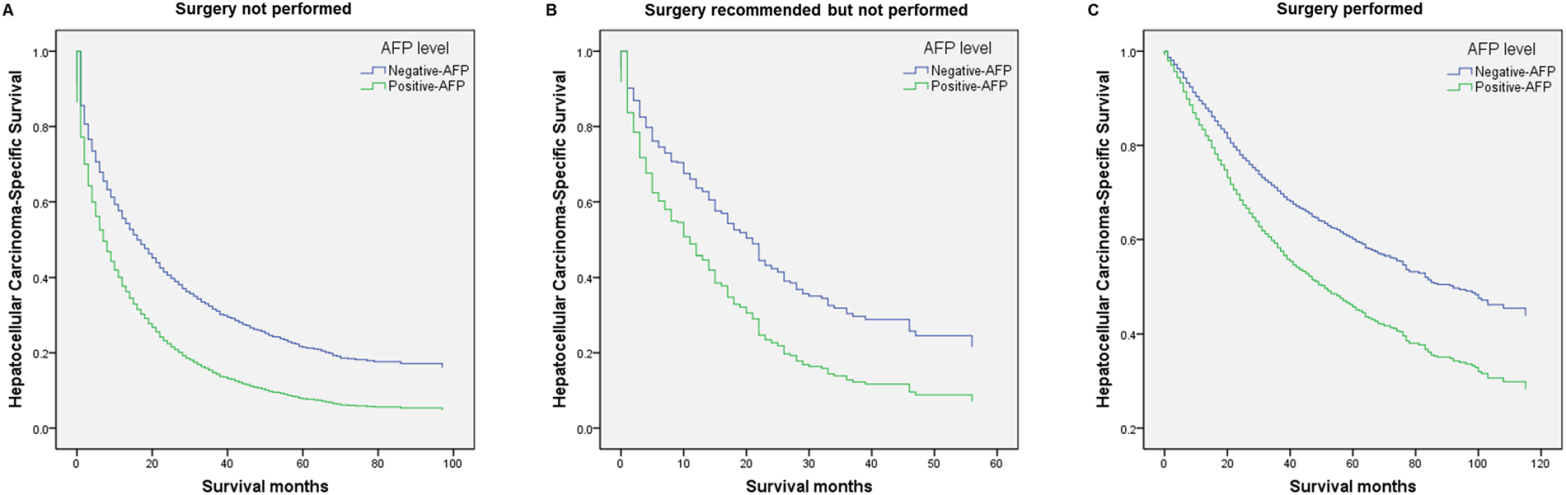
Survival curves generated using Cox models according to AFP level. Survival curves generated using Cox models that compare the association of “negative-AFP” and “positive-AFP” with hepatocellular carcinoma-specific survival of (**A**) patients who had not undergone surgery, (**B**) patients for whom surgery was recommended but not performed, and (**C**) patients who had undergone surgery.

Univariate analysis of 5931 patients using the Kaplan–Meier method was conducted to evaluate the association between AFP levels and survival of patients with HCC who did not undergo surgery. Sex (*P* = 0.002), race (*P* < 0.001), degree of fibrosis (*P* = 0.022), AFP level (*P* < 0.001), and marital status (*P* < 0.001) were identified as significant risk factors for poor survival. Multivariable Cox regression analysis identified sex (HR, 0.883; 95% CI, 0.819–0.952; *P* = 0.001), degree of fibrosis (HR, 0.880; 95% CI, 0.808–0.959; *p* = 0.004), AFP level (HR, 1.660; 95% CI, 1.534–1.797; *P* < 0.001), marital status (HR, 1.133; 95% CI, 1.066–1.203; *P* < 0.001), and race as independent predictors of survival (Fig. 3A and Supplementary Table S1).

A second analysis evaluated the relationship between AFP levels and HCSS of 1277 patients who met the initial eligibility criteria for patients with HCC recommended for surgery that was not performed. Kaplan–Meier analyses revealed that patients with AFP-negative had better 1-year, 3-year, and 5-year HCSS (*P* < 0.001) compared with those with AFP-positive (Fig. 2B and Table 5).

Further analysis of 296 patients was conducted to evaluate the association between AFP levels and HCSS of patients recommended for surgery that was not performed. Univariate analysis performed using Kaplan–Meier methods identified the degree of fibrosis (*P* = 0.002) and AFP levels (*P* < 0.001) as significant risk factors for poor survival. Multivariable Cox regression analysis identified the degree of fibrosis (HR, 0.617; 95% CI, 0.451–0.843; *P* = 0.002) and AFP levels (HR, 1.728; 95% CI, 1.262–2.365; *P* < 0.001) as independent predictors for survival (Fig. 3B and Supplementary Table S2).

A third analysis of 8477 patients with HCC who met the initial eligibility criterion of undergoing surgery was conducted to evaluate the association between AFP levels and HCSS. Kaplan–Meier analysis revealed that patients with AFP-negative had better 1-year, 3-year, and 5-year HCSS (*P* < 0.001) compared with those with AFP-positive (Fig. 2C and Table 5).

A continued analysis of 3065 patients was conducted to evaluate the association between AFP levels and HCSS of patients who underwent surgery. Age (*P* = 0.002), race (*P* < 0.001), AFP levels (*P* < 0.001), and marital status (*P* < 0.001) were identified as significant risk factors for poor survival. Multivariable Cox regression analysis identified age (HR, 1.254; 95% CI, 1.118–1.406; *P* < 0.001), AFP level (HR, 1.534; 95% CI, 1.348–1.745; *P* < 0.001), marital status (HR, 1.284; 95% CI, 1.142–1.443; *P* < 0.001), and race as independent predictors of survival (Fig. 3C and Supplementary Table S3).

## Discussion

We show here that the AFP level was an independent risk factor associated with tumor differentiation, TNM stage, tumor size, and survival of patients with HCC. AFP-positive was associated with less differentiated tumors, more advanced TNM stage, larger tumors, and inferior survival compared with AFP-negative.

Previous studies found that elevated AFP levels are associated with higher pathological grade^12,13^, more advanced Barcelona Clinic Liver Cancer stage^14^, TNM stage^15^, and larger tumors^15,16^. These discoveries are similar to the present results, although the former may be limited by the relatively small number of subjects. Pathological grade^17–19^, TNM-7 stage^20–23^, and tumor size^23,24^ are independent risk factors for survival, suggesting that patients with HCC with AFP-positive experience relatively poor survival. The findings of the present study support these conclusions and verify that the AFP level is an independent and negative prognostic factor for survival of patients with HCC who did not undergo surgery, including those recommended for surgery.

Moreover, the present study shows that even after liver resection, the AFP level at diagnosis was an independent risk factor for survival. The results show that the OR value of survival (1.534) of patients who underwent surgery did not significantly decrease compared with those who did not undergo surgery (1.660) or for those recommended for surgery that was not performed (1.728). These findings suggest that surgery may not significantly reverse the adverse effect of AFP-positive on HCC. Therefore, postoperative adjuvant therapy may be important for managing HCC patients with AFP-positive, which is consistent with the results of previous studies^13,25^.

Further, the present study reveals that AFP-positive was associated with worse 5-year HCSS compared with AFP-negative, which is inconsistent with previous findings^26,27^. For example, a randomized clinical trial found that the preoperative AFP level does not correlate with postoperative survival of patients with HCC, likely because of the heterogeneity of tumor stages^26^. Further, another study found that the AFP level does not have prognostic significance for patients with HCC with small tumors(≤3-cm diameter) who were treated with curative intent (liver resection, liver transplantation, radiofrequency thermal ablation, percutaneous ethanol injection). Because of the heterogeneity among therapies with curative intent, the associations require further analysis^27^.

AFP is associated with oncogenic effects. For example, AFP promotes cell proliferation^28^. Moreover, AFP stimulates cell motility and invasive growth of some HCC cell lines *in vitro* as well as the formation of metastases in a mouse xenograft model^29^. Therefore, recurrence and metastases may be more frequently associated with AFP-positive than AFP-negative. The patients with AFP-positive with unfavorable tumor phenotypes may explain why AFP-positive did not confer a survival benefit after surgery compared with patients with AFP-negative.

The present study is limited by the lack of data in the SEER HCC database for adjuvant therapy, comorbidities, recurrence, and the positive or negative levels of AFP. In conclusion, to our knowledge, to date, the present study of SEER data collected over 25 years represents the most comprehensive clinical analysis of AFP levels and reveals that the AFP level is an independent risk factor associated with pathological grade, progression, and survival of patients with HCC. Thus, AFP-positive was associated with higher pathological grades, more advanced TNM-7 stage, larger tumors, and inferior survival compared with AFP-negative, suggesting that oncologists should follow patients with HCC more closely and adjust treatment of those with AFP-positive as required. Further, patients with AFP-positive may require individualized adjuvant therapy after surgery vs those with AFP-negative. The present study suggests it may be necessary for the continued use of AFP.

## Methods

### Patient selection in the SEER database

The SEER database maintained by the United States National Cancer Institute is an authoritative source of information on cancer incidence and survival and the largest registry of cancer patients in the US, comprising approximately 30% of the country’s population^30^. In the SEER database, AFP test data acquired from medical records of patients with HCC is recorded as AFP-negative, AFP-positive, and other uncertain or unknown information. The fibrosis (or Ishak) score^31^, is an indicator of underlying liver disease with prognostic significance. The SEER database classifies fibrosis according to scores defined by the American Joint Committee on Cancer (AJCC), ranging from 0 to 4 (undetectable to moderate fibrosis), defined as “F0”, and 5 to 6 (severe fibrosis or cirrhosis), defined as “F1”. Here we used the degrees of liver fibrosis defined as “F0” and “F1.

The study cohort was assembled using data associated with HCC (1988–2013) from the 18 SEER incidence registries of Research Data + Hurricane Katrina Impacted Louisiana Cases. We initially identified 105,806 patients who matched Site recode ICD-O-3/WHO 2008 = liver and Behavior recode = Malignant for liver cancer. This number was reduced to 78,743 by selecting those with Histologic Type ICD-O-3 (codes 8170, 8171, 8172, 8173, 8174, or 8175) and then to 33,820 by further selecting those with AFP tests at diagnosis recorded as AFP-negative or AFP-positive. AFP-negative and AFP-positive were documented for 9181 (23.7%) and 29,639 (76.3%) patients, respectively. Sex and age data were available for 38,820 patients, and race information was available for 38,674. Pathological grade, TNM-7 stage, tumor size, degree of fibrosis, and marital status data were available for 14,007, 17,102, 32,102, 10,714, and 37,381 patients, respectively (Table 1). Of patients with sufficient data for sex, age, race, degree of fibrosis, AFP level, and marital status, 3796, 5574, and 9336 had data for pathological grade, TNM-7 stage, and tumor size, respectively. The data for pathological grade, TNM-7 stage, and tumor size were analyzed using the χ^2^ test, and logistic regression analysis was used to evaluate their associations with AFP-negative or AFP-positive.

Kaplan–Meier survival analysis was performed to evaluate AFP levels associated with the variables as follows: (i) surgery not performed; (ii) surgery recommended but not performed; and (iii) surgery performed. The number of patients included in each category depended on whether patients met the criteria as follows: First, we excluded patients <18 years of age at diagnosis; or those with multiple primary cancers, of which the HCC was not the first; or those with an unknown cause of death or an unknown duration of survival. This left 25,340, 1277, and 8477 concordant patients, respectively, in the categories described above (Fig. 1). Patients who did not undergo surgery were required to meet one of the inclusion criteria as follows: (i) surgery not recommended; (ii) surgery not recommended, contraindicated because of other conditions; (iii) surgery not performed, patient died before the recommended surgery; (iv) surgery recommended but not performed, because the patient refused; or (v) surgery recommended but not performed, reason unknown. Patients who were recommended for surgery but refused were required to meet one of the following criteria as follows: (i) surgery recommended but not performed, because the patient refused; or (ii) surgery recommended but not performed, reason unknown.

Univariate and multivariable survival analyses were performed to evaluate the associations of AFP levels with survival of HCC patients who did not undergo surgery, those recommended for surgery that was not performed, or those who underwent surgery. Before performing these analyses, we excluded patients >18 years of age upon diagnosis; or those with multiple primary cancers, of which the HCC was not the first; or those with an unknown cause of death or unknown time of survival. Further inclusion criteria were as follows: sufficient information for sex, age, race, fibrosis, AFP levels, and marital status. Multivariable survival analyses included 5931 patients with HCC who had not undergone surgery, 296 recommended for surgery that was not performed, and 3065 who underwent surgery (Fig. 1).

### Statistical Analysis

Patients were classified as “married,” or “non-married.” “Non-married” included widowed, never married, divorced, separated, unmarried, and domestic partner. Other races other than White or Black included American Indian/Alaska Native, and Asian/Pacific Islander. TNM stages were assigned according to the criteria described in the AJCC Cancer Staging Manual (7th Edition).

A primary focus of the present study was HCC-specific survival (HCSS), which was determined from the dates of diagnosis of HCC and HCC cause-specific death. Deaths attributed to HCC were treated as events and deaths from other causes as censored observations. The χ^2^ test was used to compare the characteristics of the “AFP-negative” and “AFP-positive” groups, well/moderately, and poorly differentiated/anaplastic tumors, TNM-7 stages I/II and III/IV, and tumors ≤5 cm or >5 cm in diameter. Binary logistic regression analysis was used to evaluate the associations of AFP levels with pathological grade, TNM-7 stage, and tumor size.

Differences in survival were assessed using two-sided Kaplan–Meier log-rank tests of the characteristics of patients with HCC who did not undergo surgery, who were recommend for surgery that was not performed, or those who underwent surgery. Multivariable Cox regression analyses were conducted to estimate the association of AFP levels with survival of patients with HCC who did not undergone surgery, for those recommended for surgery that was not performed, or for those who underwent surgery. All statistical analyses were performed using SPSS for Windows, version 22.0 (IBM, Armonk, NJ, USA).

## Electronic supplementary material

the Supplementary Information
