# Supplementary material for: The prognostic correlation of AFP level at diagnosis with pathological grade, progression, and survival of patients with hepatocellular carcinoma
Source: Sci Rep. 2017 Oct 9;7:12870. doi: 10.1038/s41598-017-12834-1 (PMC5634482; doi:10.1038/s41598-017-12834-1)
Supplement: Supplementary file 1 — the Supplementary Information [file 41598_2017_12834_MOESM1_ESM.pdf]

The prognostic correlation of AFP level at diagnosis with pathological grade, progression, and survival of patients with hepatocellular carcinoma

Dou-Sheng Bai[1*], Chi Zhang[1*], Ping Chen[1], Sheng-Jie Jin[1], Guo-Qing Jiang[1]

[1]Department of Hepatobiliary Surgery, Clinical Medical College of Yangzhou University, Yangzhou 225001, China

*These authors are contributed equally to the work.

Correspondence and requests for materials should be addressed to G.Q.J. (mail: jgqing2003@hotmail.com).

Table S1. Univariate and multivariable survival analysis of the association of AFP levels with the specific survival of patients with HCC who did not undergo surgery.

Variable	Total	3-year HCCS	5-year HCCS	Univariate analysis		Multivariable analysis	
	5931			Log rank χ2 test	*P*	HR (95%CI)	*P*
Sex				9.599	0.002		0.001
Male	4705	17.5%	10.5%			Reference	
Female	1226	22.2%	10.5%			0.883 (0.819–0.952)	
Age				0.001	0.971		NI
<60	2764	18.5%	11.5%				
≥60	3167	18.4%	9.8%				
Race				30.916	<0.001		<0.001
White	4117	16.8%	9.4%			Reference	
Black	747	15.8%	8.4%			0.985 (0.901–1.078)	0.747
Other*	1067	26.2%	16.5%			0.821 (0.756–0.890)	<0.001
Fibrosis				5.248	0.022		0.004
F0†	796	17.7%	9.9%			Reference	
F1††	5135	18.5%	10.8%			0.880 (0.808–0.959)	
AFP				169.657	<0.001		<0.001
Negative	1234	29.6%	17.6%			Reference	
Positive	4697	15.6%	8.9%			1.660 (1.534–1.797)	
Marital Status				22.971	<0.001		<0.001
Married	2907	20.3%	12.6%			Reference	
Non-married#	3024	16.6%	8.5%			1.133 (1.066–1.203)	

Abbreviations: HCSS, hepatocellular carcinoma-specific survival.

*Other includes American Indian/Alaska native, and Asian/Pacific Islander.

†F0, equivalent to Ishak score 0–4.

††F1, equivalent to Ishak score 5–6.

#Non-married includes widowed, never married, divorced, separated, unmarried, and domestic partner.

NI: not included in the logistic multivariable regression analysis.

Supplementary Table S2. Univariate and multivariable survival analysis of the association of AFP level on specific survival of patients with HCC who were recommend for surgery that was not performed.

Variable	Total	3-year HCCS	5-year HCCS	Univariate analysis		Multivariable analysis	
	296			Log rank χ2 test	*P*	HR (95% CI)	*P*
Sex				1.337	0.247		NI
Male	255	16.0%	12.2%				
Female	41	26.5%	12.1%				
Age				0.971	0.324		NI
<60	121	18.1%	18.1%				
≥60	175	17.7%	3.8%				
Race				5.602	0.061		NI
White	206	14.6%	12.3%				
Black	52	24.5%	10.2%				
Other*	38	25.8%	0%				
Fibrosis				9.639	0.002		0.002
F0†	57	11.2%	5.6%			Reference	
F1††	239	19.1%	11.3%			0.617 (0.451–0.843)	
AFP				12.389	<0.001		<0.001
Negative	74	31.5%	0%			Reference	
Positive	222	13.2%	11.0%			1.728 (1.262–2.365)	
Marital Status				1.043	0.307		NI
Married	102	20.8%	10.4%				
Non-married#	194	15.8%	13.1%				

Abbreviations: HCSS, hepatocellular carcinoma-specific survival.

*Other includes American Indian/Alaska native, and Asian/Pacific Islander.

†F0, equivalent to Ishak score 0–4.

††F1, equivalent to Ishak score 5–6.

#Non-married includes widowed, never married, divorced, separated, unmarried, and domestic partner.

NI: not included in the logistic multivariable regression analysis.

Supplementary Table S3. Results of univariate and multivariable survival analysis to evaluate the influence of AFP level on specific survival of HCC patients for whom surgery was performed

Variable	Total	3-year HCCS	5-year HCCS	Univariate analysis		Multivariable analysis	
	3065			Log rank χ2 test	*P*	HR (95%CI)	*P*
Sex				0.535	0.465		NI
Male	2382	62.8%	51.2%				
Female	683	59.9%	48.9%				
Age				9.802	0.002		<0.001
<60	1544	64.3%	54.2%			Reference	
≥60	1521	59.6%	46.3%			1.254 (1.118–1.406)	
Race				18.453	<0.001		0.003
White	1968	62.0%	51.2%			Reference	
Black	327	53.7%	35.5%			1.238 (1.037–1.477)	0.018
Other*	770	65.8%	55.0%			0.871 (0.758–1.001)	0.051
Fibrosis				0.001	0.972		NI
F0†	820	63.7%	51.1%				
F1††	2245	61.5%	50.5%				
AFP				43.180	<0.001		<0.001
Negative	1016	70.4%	60.0%			Reference	
Positive	2049	58.0%	46.2%			1.534 (1.348–1.745)	
Marital Status				22.509	<0.001		<0.001
Married	1917	65.5%	54.6%			Reference	
Non-married#	1148	56.3%	44.0%			1.284 (1.142–1.443)	

Abbreviations: HCSS, hepatocellular carcinoma-specific survival.

*Other includes American Indian/Alaska native, and Asian/Pacific Islander.

†F0, equivalent to Ishak score 0–4.

††F1, equivalent to Ishak score 5–6.

#Non-married includes widowed, never married, divorced, separated, unmarried, and domestic partner.

NI: not included in the logistic multivariable regression analysis.
